# Rib Plating as an Effective Approach to Managing Traumatic Rib Injuries: A Review of the Literature

**DOI:** 10.7759/cureus.29664

**Published:** 2022-09-27

**Authors:** Christopher Adereti, Jamesa Fabien, Jeanette Adereti, Muller Pierre-Louis, Daniel Chacon, Vincent Adereti

**Affiliations:** 1 Surgery, Ross University School of Medicine, Bridgetown, BRB; 2 Medicine, Ross University, Bridgetown, BRB; 3 Surgery, Texas Tech University, Lubbock, USA

**Keywords:** thoracic trauma, flail chest, rib fixation, surgical stabilization, surgical outcomes, rib plating

## Abstract

Timely repair is essential to maximizing outcomes in patients with traumatic rib injuries, whether in the presence or absence of flail chest (FC) or existing as single or multiple rib fractures (MRF), due to its high morbidity and mortality rate. This review focuses primarily on the plating system as an effective surgical approach to stabilizing these injuries.

Literature was surveyed using the Google Scholar, PLOS One, and PubMed search engines between August 2021 and April 2022. A total of 34 articles were included herein, and primary and secondary outcomes were assessed. The primary outcomes of interest were intensive care unit length-of-stay (ICU LOS), hospital length-of-stay (HLOS), ventilatory requirements, and mortality rate. The secondary outcomes of interest were postoperative pain level and postoperative complications.

The majority of the studies included herein reported lower ICU LOS, HLOS, and ventilation requirements in surgical patients when compared to conservatively managed patients. However, variables such as the presence or absence of FC also impacted outcomes in certain studies. Mortality rate and postoperative pain were largely underreported in the selected studies, but limited data from these studies suggest that these outcomes tend to be lower in surgical patients compared to those treated conservatively. When present, postoperative complications were often less severe amongst surgical patients compared to conservatively managed patients. Results further suggest that surgical repair is associated with lower pain severity as early as 72 hours postop. Likewise, findings suggest that early rib fracture stabilization is superior to late stabilization and often yields a sooner return to a baseline health status.

Few studies report little to no statistical difference in primary and secondary outcomes between operative and conservative treatment. However, there is greater evidence that suggests the contrary, with better short-term and potential for better long-term outcomes in patients who undergo rib fixation.

## Introduction and background

The rate of mortality secondary to chest trauma is estimated to be 20%-25%, with elderly adults succumbing to their injuries more often than younger patients [1-3]. Rib fractures, the most common traumatic thoracic injury have long been a significant cause for concern due to their high incidence in the general population (20%-39%) and associated morbidity and mortality (10%-15%) [4,5]. Common complications of rib fractures include pulmonary contusions, flail chest (FC), and atelectasis, which can lead to acute respiratory failure (ARDS) [6]. Rib fractures are also associated with life-threatening conditions such as pneumothorax, hemothorax, and blunt cardiac injury. Patients who do recover from rib fractures can be affected for several months after the injury [7]. Despite this knowledge, the standard approach to treating rib injuries has traditionally been via conservative management with pain control optimization, use of positive pressure ventilation, pulmonary hygiene, chest physiotherapy, and frequent mobilization [3,8,9]. Rib fractures managed conservatively can suffer gradual displacement during the recovery phase and thus yield deformity, degeneration, and respiratory complications [6,10]. Current literature suggests that, when indicated, conservative management has generally been less effective than surgical stabilization in managing these patients long-term and therefore resulted in substantial hospital and societal costs [11].

Many studies in the literature suggest that rib stabilization using a plating system has shown the potential in reducing immediate and long-term complications of rib fractures, including the development of pneumonia, the need for tracheostomy, prolonged hospitalization, increased dependence on pain medication, and mortality from FC. Currently, there are multiple indications and contraindications for rib fracture stabilization as outlined in the 2020 guidelines by the Chest Wall Injury Society (CWIS) [12].

Indications include chest wall instability: either FC, the presence of at least three consecutive ribs broken in two locations, or three consecutive bi-cortically displaced rib fractures [4,12]. This is applicable to both ventilated and non-ventilated patients [12]. The procedure has also been used in ventilated patients who fail to wean from the ventilator with or without FC, non-ventilated patients with three or more severely displaced acute rib fractures in ribs 3-10 in combination with two or more pulmonary physiologic derangements despite loco-regional anesthesia and multi-modal pain therapy [12]. Further indications for rib fracture stabilization include patients with implosion chest wall injuries (i.e., stoved-in chest), patients undergoing thoracotomy for another indication such as hematoma evacuation, or patients with greater than 30% loss of chest volume [10,12,13]. However, it is recommended that these indications be considered on a case-by-case basis due to limited support for their use in various studies [12].

Absolute contraindications include shock or incomplete resuscitation, fractures outside of ribs 3-10, severe traumatic brain injury (TBI), and acute myocardial infarctions [12,14]. Relative contradictions include age less than 18 and significant co-morbidities such as cardiopulmonary disease, mild to moderate TBI, active malignancy, spinal cord injury, empyema, or a history of chest wall radiation [12].

**Figure 1:**
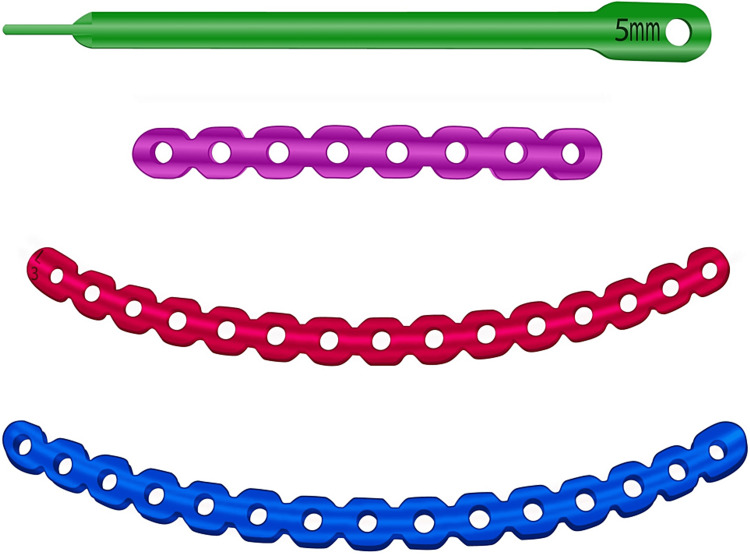
Illustration displaying an intramedullary splint (top) and three Synthes MatrixRIB plates.

**Figure 2:**
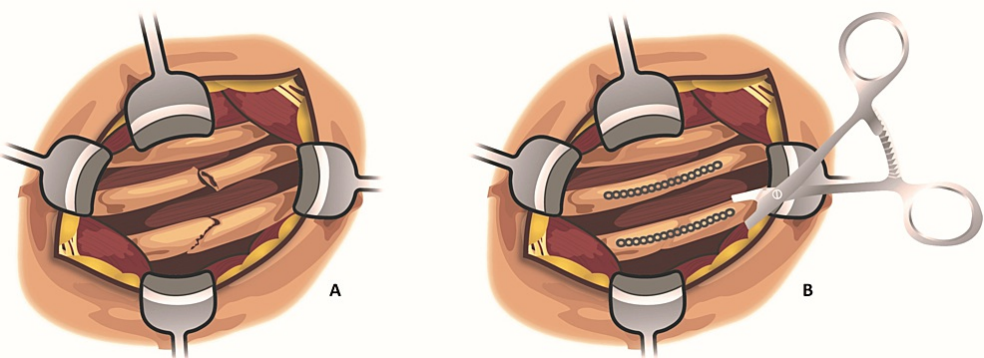
Illustrations displaying multiple rib fractures before (A) and after surgical stabilization with plating application (B).

The plating system uses titanium plates that individually conform to patients’ ribs and protect against instability and nonunion (Figures 1, 2A, 2B). Though rib plating has the potential to modernize the management of rib fractures, it remains a second-line treatment for conservative management. A survey conducted by Mayberry and colleagues in 2009 revealed that rib plating was underutilized because the published literature on the technique was sparse and unfamiliar to most surgeons [15]. In 2014, Dehghan et al. performed a retrospective analysis on traumatic FC injuries using the National Trauma Data Bank which showed that only 0.7% of patients were treated with surgical fixation of the chest wall [9]. Although lack of procedural familiarity may explain this finding to some extent, the researchers cite the years in which the study population (2007-2009) was selected as a possible reason for this finding with fewer rib fixation procedures likely occurring during those years [9]. Herein, we focus primarily on the plating system as an effective surgical approach to stabilizing patients with traumatic rib injuries.

## Review

Search strategy

We aimed to identify the use of rib plating as an effective surgical procedure for the management of traumatic rib injuries compared to the current standard of care in which patients are managed conservatively with pain control optimization, use of positive pressure ventilation, pulmonary hygiene, and frequent mobilization. For the purposes of this study, the efficacy of rib plating is defined by primary outcomes: ICU LOS, HLOS, ventilatory requirements, and mortality rates, and secondary outcomes: postoperative pain level and postoperative complications. 

A literature search in the PubMed, Google Scholar and PLOS One databases was conducted between August 2021 and April 2022 using the following Medical Subject Heading terms: “rib plating”, “rib stabilization”, and “surgical rib fixation”. The search was limited to the English language with publication dates between January 2005 and December 2021 (one exception was a randomized control study [RCT] by Tanaka et al.). Only relevant articles with free full access were included in our review while case reports, case series (except for an article by Wijffels et al.), systematic reviews, and “abstract-only” articles were excluded. The authors independently reviewed potentially eligible titles and abstracts and contributed to the review process. Disagreements between the reviewers were infrequent and resolved by majority consensus. Where applicable, articles not meeting the inclusion criteria were then excluded from the study.

**Figure 3:**
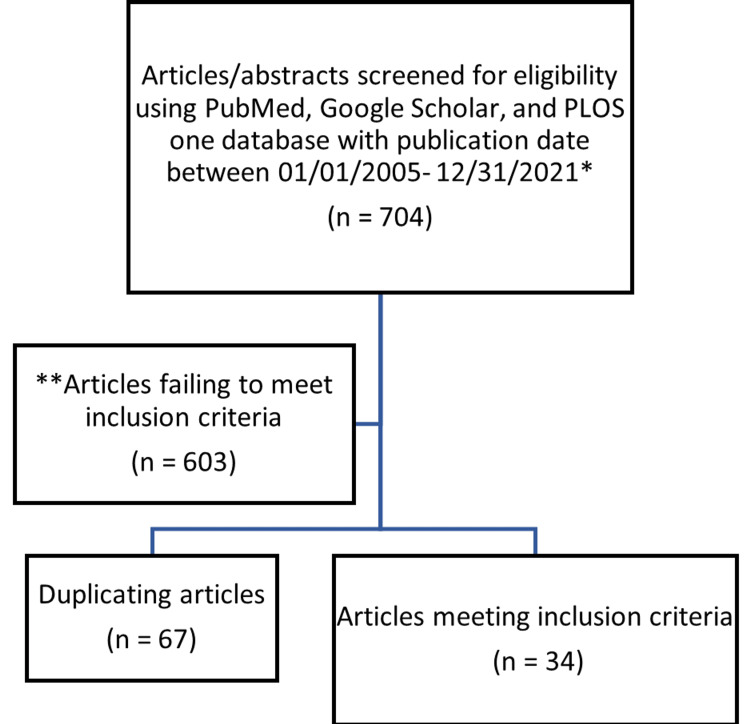
Outline of the article selection process. *Exception: article by Tanaka et al. (2002). **Exception: case series by Wijffels et al. (2020).

A total of 704 titles and abstracts from the PubMed, Google Scholar, and PLOS One databases were assessed for eligibility. Non-relevant articles were excluded which narrowed our search to 101 articles. Of these, 67 articles were repeated and were excluded, and the remaining 34 papers were assessed in detail for study inclusion (Figure 3).

Results

Few RCTs demonstrating the efficacy of rib fixation exist. In one such study by Tanaka et al., 37 FC patients were randomized into a conservative group (n = 19) or surgical group (n = 18) five days post-injury [16]. Indications for mechanical ventilation included the presence of hypoxia and/or hypercarbia (PaO_2_ < 60 mm Hg, PaCO_2_ > 50 mm Hg) under 40% inspired oxygen inhalation, associated severe trauma with unconsciousness and/or shock state, and presence of airway obstruction or repeated atelectasis [16]. Both patient groups were managed equally leading up to surgery. Outcomes revealed a shorter ventilatory period (10.8 ± 3.4 days vs. 18.3 ± 7.4 days, p < 0.05), trauma intensive unit stay (16.5 ± 7.4 days vs. 26.8 ± 13.2 days, p < 0.05), postop pain at 12 month follow up (39% vs. 89%, p < 0.05), and lower incidence of pneumonia at three weeks post injury (22% vs. 90%, p < 0.05) in the surgical group [16].

In another RCT by Granetzy et al., 40 patients with FC were randomized for conservative management (using strapping, packing, and mechanical ventilation) or surgical treatment [10]. Patients with head trauma with disturbed conscious level, associated injuries such as myocardial contusion, severe trauma to other systems, and fractures of the upper three ribs only, were excluded from the study [10]. All surgical patients were operated on within 24-36 hours after admission. Prior to this, these patients were managed similarly to the conservative group [10]. After management of both groups, the researchers found that chest wall stability occurred in 85% of surgical patients compared to 50% in the conservatively treated group [10]. Primary outcomes were lower in the former group compared to the latter (p < 0.001), except for the mortality rate which was non-significant between the two groups [10].

In a third RCT by Marasco et al., 46 mechanically ventilated patients with FC were randomized to a surgical group or conservative group [17]. Like Tanaka et al. and Granetzy et al., results showed lower total ICU stay (324 (238 - 380) hours vs. 448 (323 - 647) hours, p = 0.03) in surgically treated patients compared to conservatively managed patients [17]. Furthermore, the surgical group required less ventilation post-extubation (3 (0 - 25) hours vs. 50 (17 - 102) hours) [17]. Postoperative pain levels were not specifically assessed and complications such as pneumonia were not statistically significant between the two groups (48% vs. 74%, p = 0.07) [17].

In a more recent RCT by Pieracci et al., the researchers performed a multicenter study assessing the efficacy of rib fracture stabilization in patients with non-FC (NFC) injuries [18]. This study represents the first prospective, multicenter trial focused on the stabilization of NFC. Patient inclusion criteria were centered on CWIS guidelines. The presence of FC, age < 18 or ≥ 80 years, moderate to severe TBI, and enrollment > 72 hours post injury were amongst the exclusion criteria [18]. The initial sample size consisted of 848 patients; however, 87% of patients failed screening resulting in an enrollment of 110 patients. The most common reasons for screening failure were fewer than three displaced rib fractures (30.3%) and less than 50% displacement of fractures (21.3%) [18]. Patients who failed screening, as compared those who were enrolled, were more likely to be female (34% vs. 25%, respectively), older (58 vs. 55 years, respectively), and have a lower injury severity score (ISS 9 vs. 14, respectively) [18]. Results of the study showed no difference in ICU LOS, HLOS, or median ventilator days between the surgical and conservative groups and no mortality occurred in either group [18]. Surgical management was associated with lower numeric pain scores (NPS) as early as hospital day 7 (4.7 vs. 6.3, p < 0.01) [18]. This held true at week two follow up (2.9 vs. 4.5, p < 0.01), and at four weeks and eight weeks follow up (2.4 vs. 3.3, p < 0.03) and (1.5 vs. 3.3, p <0.02), respectively. NPS on hospital days 1-6 were non-significant [18]. Pleural space complications were found to be lower in the surgical group compared to the conservative group (0% vs. 10.2%, p = 0.02) while pneumonia rates were non-significant between the two groups (2.0% vs. 6.7%, p = 0.37) [18]. Overall, results from this study underscore a more modest benefit to rib fixation in the NFC population compared to those seen amongst the FC populations in previous RCTs.

In a study conducted by Taghavi et al., 114,972 patients with rib fractures were classified as having FC (n = 5,106), multiple rib fractures (MRF) (n = 85,140), or a single rib fracture (SRF) (n = 24,726) [19]. The median age (in years) of patients in each group was 57.0, 56.0, and 50.0, respectively. Females comprised 24.9% of participants with FC, 32.3% of those with MRF, and 31.2% of those with SRF [19]. In total, 98.6% of study participants were treated conservatively while 1.4% were treated surgically. Amongst in-hospital patients, those who underwent surgical stabilization were found to require longer HLOS (5.0 vs 13.0 days; p < 0.001), ICU LOS (4.0 vs 8.0 days; p < 0.001), mechanical ventilation (19.5% vs. 40.6%; p < 0.001) and develop higher rates of ARDS (3.1% vs. 1.0 %; p < 0.001) compared to patients treated conservatively [19]. However, the authors posit that this is likely attributed to a marker of severe injury as these patients were more likely to have FC and high ISS [19]. Mortality rates, however, were lower in patients who underwent surgical stabilization compared to patients managed conservatively (2.5% vs. 4.8%, p < 0.001) [19]. Similar findings were reported in a retrospective cohort study by Farquhar and colleagues [20]. The authors reported that the conservatively managed group had significantly better outcomes than the surgical group regarding ventilation requirements (3.1 vs. 6.1 days, p = 0.012), ICU LOS (3.7 vs. 7.4 days, p = 0.009), total HLOS (16.0 vs. 21.9 days, p = 0.044) and rates of pneumonia (22% vs. 63%, p = 0.004) [20]. However, there were no significant differences in long-term complications, such as chest pain or dyspnea [20].

Conversely, other studies have reported improved clinical outcomes in patients undergoing rib plating compared to conservative management. In their retrospective analysis, Liu et al. evaluated the clinical effects of MRF treatments using rib plating and found that the surgical group experienced fewer ICU LOS and HLOS than the conservative group (4.02 ± 1.41 vs. 5.06 ± 1.80 days, p = 0.001) and (13.12 ± 4.21 vs. 18.57 ± 5.39 days, p < 0.001), respectively [21]. In a second study by Xu et al., better short-term outcomes were observed in the surgery group, such as total mechanical ventilation time (10.5 ± 3.7 vs. 13.7 ± 4.4 days, p = 0.03), ICU LOS (15.9 ± 5.0 vs. 19.6 ± 5.0 days, p = 0.05), and Acute Physiology and Chronic Health Evaluation (APACHE II) scores on the 14th day (6.5 ± 3.8 vs. 10.1 ± 4.7, p = 0.02) [5]. Comparable results were echoed in a study by Zhang et al., who found fewer mechanical ventilation needs, shorter ventilation time, shorter HLOS, lower incidence of respiratory complications and thoracic deformity, and improved pulmonary function in post-surgical patients compared to those treated conservatively [22].

Yet, another study reported that HLOS, ICU LOS, duration of mechanical ventilation, and mortality were higher in the conservative group compared with those managed surgically (p < 0.001, p < 0.001, p < 0.001, and p = 0.027, respectively) [23]. However, there was no statistical difference in complications such as pneumonia or sepsis between patient groups [23]. In a study by Griffard et al., the authors matched the outcomes between operative and conservative management and found no difference in the prevalence of pneumonia (p = 0.1416) or severe ARDS (p = 0.999) between groups [24]. Furthermore, there was no significant difference between the operative and conservative groups in ventilator days (p = 0.641) or hospital days with 11 days vs. 9 days (p = 0.1358) [24]. Patients undergoing rib plating experienced longer ICU LOS at six days (IQR 4 to 9) compared with 3.5 days (IQR 2 to 9) in conservatively managed patients (p = 0.0217) [24]. Jayle et al. found no significant differences between groups for matched data and prognostic scores: ISS, revised trauma score, and trauma injury severity score [25]. However, ventilator time (142 ± 224 vs 74 ± 125 hours, p = 0.026) and overall HLOS (142 ± 224 vs 74 ± 125 hours, p = 0.026) were significantly lower for the surgical group after adjustment on prognostic scores [25]. Beks et al. found that rib fixation was not associated with ICU LOS (for FC patients) nor with HLOS (for MRF patients) while Olsén et al. and Farquhar et al. reported that there were no significant long-term differences between patients treated surgically and conservatively [20,26,27].

When comparing postoperative pain relief between surgical and conservative patients, Ağababaoğlu and Ersöz reported that pain scores were statistically significantly different in favor of the surgical group compared to the conservative treatment group (p = 0.0038 and p = 0.044, respectively) [23]. In a prospective study assessing the impact of plating in patients with rib fractures, the authors evaluated 67 patients over one year and classified them into conservative and operative groups based on a pain scale in which those with levels 5, 6, or 7 pain received conservative management, while patients with levels 8, 9, or 10 received operative management [28]. Results showed that patients undergoing surgical stabilization with plating had reduced pain and duration of disability and an increased return to work compared to the conservative group [28]. In their study aimed at investigating the curative effect of surgical treatment for 39 patients with severe NFC rib fractures, Zhang et al. found that these patients had better pain control and quality of living following surgery compared to the 39 patients that received conservative treatment with analgesia [29]. This held true at 72 hours, one week, two weeks, four weeks, six weeks, three months, and six months postop (p < 0.001) [29]. Caragounis et al. reported that surgical patients had significant outcome improvements at one-year postop concluding that final outcomes of surgical stabilization could not be finalized any sooner than this time period [30].

Findings in current literature

Though there have been previous studies detailing various rib plating systems and their application in the setting of FC, high-quality studies on NFC patterns are limited. It has been applied in patients with slipping rib syndrome, severe chest trauma associated with FC, concomitant lung lesion, serious alteration of the chest shape, and persistent and chronic pain that affects normal life [13,31]. Many studies suggest that this technique has been associated with earlier ambulation, shorter hospital duration, less opioid dependence, and greater patient satisfaction.

The most frequently reported post-surgical complications are related to underlying injuries from rib trauma, such as pulmonary contusion and pneumonia [7,32]. Rarer complications, including hardware infections, have also been reported to occur in 1-3% of patients [7,33]. As a result, it is necessary to drain any empyema that develops, initiate antibiotics, and remove the affected hardware once bacterial counts have lessened [7]. In contrast, if the fracture site is well healed, the hardware may be removed at the initial signs of complication [7]. Additional complications known to exist include wound hematoma, pleural effusion, post-thoracotomy pain syndrome, and osteomyelitis [10,28,32].

There are limited reports assessing the long-term outcomes of rib stabilization. One such study by Uchida et al. surveyed 20 patients (FC, n = 9; MRF, n = 11) who had undergone rib plating over a five-year period and assessed their quality of life [34]. The median follow-up duration was 47.5 months and the least desirable event occurring during the study period was irritation caused by a palpable plate in two of the participants [34]. Eighteen patients returned to baseline activity level without any complaints, two patients continued to undergo rehabilitation due to concomitant fractures of the extremities, and zero patients experienced implant-related complications requiring explantation [34].

Questions still exist regarding the effect rib stabilization has on ICU LOS. Fokin et al. and Griffard et al. observed longer ICU LOS for those who had surgical rib stabilization while a study by Xiao et al. found that surgical rib fixation shortened ICU LOS in patients with MRF and FC, but it did not in those without FC [24,35,36]. Majeed et al. found no statistical difference in HLOS or the number of days for ventilator support and findings on ICU LOS were not reported [37]. Acker et al. reported there to be no statistical difference in HLOS, ICU LOS, and mortality rates between surgically treated patients and conservatively treated patients [38]. However, there was notable clinical improvement in ventilation in patients with FC and MRF following surgical stabilization [38].

Discussions on surgical timing have been extensively covered in the literature. Many studies recommend that operative repair occurs within the first 48-72 hours post-trauma as inflammation and callus formation have yet to occur, resulting in an easier operation [12,35,39]. When comparing surgical timing in patients undergoing rib fixation within three, six, or 10 days after hospital admission, Otaka et al. found that surgical rib fixation within three days after admission was associated with a shorter duration of mechanical ventilation (percent difference, -42.9%; 95% CI, -57.4 to -23.3) and shorter HLOS (percent difference, -19.6%; 95% CI, -31.8 to -5.2) [40]. However, there were no significant differences between the groups in all-cause 28-day in-hospital mortality (risk difference, -0.02; 95% CI, -0.07 to 0.03) or in any in-hospital outcomes between those who had and had not undergone rib fixation within six or 10 days after admission [40].

Similarly, Su et al. also analyzed the impact of early (stabilization within three days of injury) versus late (stabilization after three days of injury) surgical stabilization on perioperative and clinical outcomes in 33 patients with severe rib injuries [41]. Sixteen of the patients underwent early stabilization and 17 underwent late stabilization [41]. Results showed that patients receiving early intervention had a notably shorter duration of mechanical ventilation (median = 36 vs. 90 hours, p = 0.03), ICU LOS (median = 123 vs. 230 hours, p = 0.004), and HLOS (median = 12 vs. 18 days, p = 0.03) compared to patients who underwent late surgical repair, though mortality rates were nonsignificant between the groups [41]. Descriptions of each study are listed in Table 1. Primary and secondary outcomes of the various studies are reported in Tables 2, 3, respectively.

**Table 1:** Description of studies *Data presented as median age. M - male, F - female, IRF - iatrogenic rib fractures, SSRF - surgical stabilization of rib fractures, FC - flail chest, NFC - non-flail chest, MRF - multiple rib fractures, RCT - randomized control trial, SRF - single rib fracture

Study	Research Type	Total study population (n)		Chest Wall Injury Assessed	Mean age (in years)						Ratio of M/F participants (%)			
Lin et al. (2)	Retrospective	1621		Rib fracture, sternum fracture, lung contusion, hemothorax, pneumothorax	51.2 (18-95)						M = 72.5			
P =	Not specified													
Xu et al. (5)	Retrospective	32		FC	Surgical group (36.4 ± 13.5)	Conservative group (39.0 ± 11.6)					Surgical group (37.5/0.16)			Conservative group (37.5/0.09)
P =					0.727						0.593			
Tarng et al. (6)	Retrospective	65		MRF	47.25						M = 91.6			
P =	Not specified													
Wijffels et al. (8)	Retrospective	70		FC	Surgical group (60, 40–69)				Conservative group (49, 40–63)		Surgical group (M = 65)		Conservative group (M = 79)	
P =					0.268						0.354			
Dehgan et al. (9)	Retrospective	3,467		FC	52.5						77/23			
P =					Not specified						Not specified			
Granetzy et al. (10)	RCT	40		FC	Surgical group (24 - 55)		Conservative group (12 - 60)				Surgical group (35/15)	Conservative group (42.5/7.5)		
P =					< 0.001									
Qiu et al. (11)	Retrospective	162		FC, NFC	Surgical group (FC) (34.76 ± 12.92)			Conservative group (FC) (35.53 ± 14.32)			Surgical group (FC) (15/6)		Conservative group (FC) (12/5)	
P =					0.863			0.955						
Jian et al. (13)	Retrospective	54		FC, MRF	Surgical group (51.3 ± 13.0)			Conservative group (50.0 ± 14.3)			Surgical group (33.3/13)		Conservative group (30/16.7)	
P =					0.721						1.000			
Tanaka et al. (16)	RCT	37		FC	Surgical group (43 ± 12)					Conservative group (49 ± 9)	Surgical group 12/6		Conservative group 14/5	
P =					Not statistically significant						Not statistically significant			
Marasco et al. (17)	RCT	46		FC	Surgical group (57.8 ± 17.1)					Conservative group (59.3 ± 10.4)	Surgical group (43.5/6.5)		Conservative group (43.5/6.5)	
P =					0.72						1.0			
Pieracci et al. (18) **	RCT	110		NFC	Surgical group (54.6)					Conservative group (55.3)	Surgical group (M = 76.5)		Conservative group (M = 74.1)	
	P =				0.85						0.83			
	RCT	110		NFC	Randomized subjects (55.3)					Observational subjects (54.5)	Randomized subjects (M = 86.4)		Observational subjects (72.4)	
	P =				0.09						-0.35			
Taghavi (19)	Retrospective	114,972		FC, SRF, MRF	Surgical group (55) *					Conservative group (55) *	Surgical group (57.8 ± 17.1)		Conservative group (59.3 ± 10.4)	
P =					<0.773						<0.001			
Farquhar et al. (20)	Retrospective	55		FC	Surgical group (51.3 ± 14.3)					Conservative group (56.5 ± 15.9)	Surgical group (79/21)		Conservative group (69/31)	
P =					0.42						0.54			
Liu et al. (21)	Retrospective	110		MRF	Surgical group ≥ 60 (19.1)					Conservative group ≥ 60 (22.7)	Surgical group (30.9/22.7)		Conservative group (25.5/20.9)	
					Surgical group < 60 (34.6)					Conservative group < 60 (23.6)				
P =					0.155						0.774			
Zhang et al. (22)	Retrospective	52		FC	Surgical group (57.8 ± 12.0)					Conservative group (59.5 ± 9.9)	Surgical group (40.4/15.4)		Conservative group (30.8/13.5)	
P =					>0.10						>0.05			
Ağababaoğlu & Ersöz (23)	Retrospective	63		FC	Surgical group (45.8 ± 15.6)					Conservative group (43.7 ± 12.1)	Surgical group (32.35/67.65)		Conservative group (47.62/55.81)	
P =					0.553						0.858			
Griffard et al. (24)	Retrospective	165		FC, NFC	Surgical group (59, 48 - 68)					Conservative group (59, 46.5 - 69)	Not reported			
P =					0.974						Not reported			
Jayle et al. (25)	Retrospective	20		FC	Surgical group (47.9 ± 10.6)					Conservative group (50.5 ± 12.5)	Surgical group (M = 80)		Conservative group (M = 80)	
P =					0.48						1.0			
Beks et al. (26)	Retrospective	332		FC, MRF	56 ± 17						M = 77			
P =					Not reported						Not reported			
Olsén et al. (27)	Retrospective	61		MRF	Surgical group (58.3 ± 14.6)					Conservative group (58.4 ± 16.1)	Surgical group (36.1/14.8)		Conservative group (41/8.2)	
P =					0.908						0.363			
Khandelwal et al. (28)	Prospective	61		Not specified	Surgical group (47.38)					Conservative group (45.30)	65.57/34.42			
P =					Not reported						Not reported			
Zhang et al. (29)	Retrospective	78		NFC	Surgical group (48.7 ± 9.6)					Conservative group (50.2 ± 10.1)	Surgical group (35.9/14.1)		Conservative group (37.2/7.8)	
P =					0.059						0.071			
Caragounis et al. (30)	Prospective	54		MRF, FC	57*						74/26			
P =					Not reported						Not reported			
Billè (31)	Retrospective	18		MRF, IRF, lung herniation, chest wall tumor	61*						67/33			
P =														
Beks et al. (32)	Retrospective	103		FC, MRF	FC (57, 48-69) *					MRF (56, 47-64) *	FC (M = 78)		MRF (M = 82)	
P =					Not reported						Not reported			
Thiels et al. (33)	Retrospective	122		FC, NFC	59.5 (16.4)						M = 72.9			
P =					Not reported						Not reported			
Uchida et al. (34)	Retrospective	20		FC, MRF	64 (56–73) *						60/40			
P =					Not reported						Not reported			
Fokin et al. (35)	Retrospective	174		FC, NFC	Surgical group (55.9)					Conservative group (55.4)	Surgical group (M = 74.7)		Conservative group (M = 73.6)	
P =					0.8						0.9			
Xiao et al. (36)	Retrospective	1,201		FC, MRF	Surgical group (FC) [52.1 ± 9.7]					Conservative group (FC) [49.2 ± 9.1]	Surgical group (FC) [75.2/24.8]		Conservative group (FC) [56.9/43.1]	
	P =				0.054						0.011			
	Retrospective		1,201	FC, MRF	Surgical group (MRF) [50.2 ± 10.4]					Conservative group (MRF) [49.1 ± 9.3]	Surgical group (MRF) [84.2/15.8]		Conservative group (MRF) [79.1/20.9]	
	P =				0.083						0.042			
Majeed et al. (37)	Prospective	43		MRF, FC	51.35 ± 13.75						86.05/13.95			
P =					Not reported						Not reported			
Acker et al. (38)	Prospective	85		FC, MRF	Surgical group (45.1 ± 18.6)					Conservative group (41.57 ± 17.09)	Surgical group (M = 29.2)		Conservative group (M = 24.6)	
P =					0.40						0.43			
Prins et al. (39)	Retrospective	Assessment of nine studies		Addressing optimal timing for SSRF	n/a						n/a			
Otaka et al. (40)	Retrospective	6315		FC	No surgery ≤ 3 days after admission (64.1 ± 19.8)					Surgery ≤ 3 days after admission (66.6 ± 14.6)	No surgery ≤ 3 days after admission (M = 65.0)		Surgery ≤ 3 days after admission (M = 66.1)	
P =					Not reported						Not reported			
Su et al. (41)	Retrospective	33		FC	Early surgical group (62, 19–92) *					Late surgical group (68, 19–92) *	Early surgical group (33.3/15.2)		Late surgical group (45.5/6.1)	
P =					0.47						0.24			

**Table 2:** Primary outcomes – (a) Outcomes among FC patients only. (b) Outcomes among non-FC patients only. (c) Outcome between operative and conservative management in patients with FC versus MRF. (d) Outcomes pertaining to surgical timing (i.e., <3 days vs > 3 days) as opposed to surgical versus conservative management. *Trauma intensive care unit. ICU LOS - intensive care unit length-of-stay, HLOS - hospital length-of-stay, SSRF - surgical stabilization of rib fractures, FC - flail chest, MRF - multiple rib fractures

Study	Primary Outcome	SSRF	Conservative		P value	
Xu et al. (5)	ICU LOS	15.9 ± 5.0	19.6 ± 5.0 days		p = 0.05	
	HLOS	Not reported	Not reported		Not reported	
	Ventilation Requirements	10.5 ± 3.7	13.7 ± 4.4 days		p = 0.03	
	Mortality Rate	Not specified				
Tarng et al. (6)	ICU LOS	Mean 7.33 days (SD = 0.95)	Mean 16.70 days, (SD = 9.62)		Not reported	
	HLOS	Mean 15.17 days (SD = 2.69)	Mean 35.55 days, (SD = 19.46)		Not reported	
	Ventilation Requirements	Mean 6.42 days (SD = 0.79)	Mean 11.35 days, (SD = 16.35)		Not reported	
	Mortality Rate	Not reported				
Wijffels et al. (8)	ICU LOS	5 (4–11) days	10 (3–20) days		p = 0.296	
	HLOS	20 (13–30) days	23 (14–35) days		p = 0.495	
	Ventilation Requirements	4 (2–9) days	12 (6–18) days		p = 0.011	
	Mortality Rate	Not reported				
Granetzny et al. (10)	ICU LOS	Mean 9.6 days	Mean 14.6 days		p <0.001	
	HLOS	Mean 11.7 days	Mean 23.1 days		p <0.001	
	Ventilation Requirements	Mean 2 days	12 days		p <0.001	
	Mortality Rate	10%	15%			Non-significant
Qiu et al. (11)^a^	ICU LOS	7.19 ± 1.67 days	10.29 ± 2.31 days		p = 0.016	
	HLOS ^b^	11.09 ± 1.88 days	15.93 ± 2.75 days		p = 0.013	
	Ventilation Requirements	5.71 ± 1.35 days	9.06 ± 3.58 days		p = 0.005	
	Mortality Rate ^b^	4.76%	11.76%		p = 0.491	
Jian et al. (13)	ICU LOS^ a^	5.9 ± 0.6 days	10.6 ± 1.9 days		p = 0.000	
	HLOS	11.5 ± 1.9 days	13.9 ± 4.0 days		p = 0.006	
	Ventilation Requirements^ a^	4.5 ± 0.7 days	7.9 ± 1.7 days		p = 0.000	
	Mortality Rate	No mortality reported in either group				
Tanaka et al. (16)	TICU LOS*	16.5 ± 7.4 days	26.8 ± 13.2 day			p < 0.05
	HLOS	Not reported				
	Ventilation Requirements	10.8 ± 3.4 days		18.3 ± 7.4 days		p < 0.05
	Mortality Rate	Not reported				
Marasco et al. (17)	ICU LOS	285 hrs [range 191 - 319 hrs]		359 hrs [range 270 - 581 hrs]		p = 0.03
	HLOS	20 days [range 18 - 28 days]		25 days [range 18 - 38 days]		p = 0.24
	Ventilation Requirements	151.8 ± 83.1 hrs		181.0 ± 130.2 hrs		p = 0.37
	Mortality Rate	0		1		p = 0.87
Pieracci et al. (18)	ICU LOS/ HLOS	No difference between groups noted				Not reported
	Ventilation Requirements	median ventilator days for each group = 0				p = 0.79
	Mortality Rate	No mortality reported in either group				
Taghavi et al. (19)	ICU LOS	4.0 days	8.0 days		p < 0.001	
	HLOS	5.0 days	13.0 days		p < 0.001	
	Ventilation Requirements	19.5%	40.6%		p < 0.001	
	Mortality Rate	2.5%	4.8%		p < 0.001	
Farquhar et al. (20)	ICU LOS	7.4 days	3.7 days		p = 0.009	
	HLOS	21.9 days	16.0 days		p = 0.044	
	Ventilation Requirements	6.1 days	3.1 days		p = 0.012	
	Mortality Rate	Not reported				
Liu et al. (21)	ICU LOS	4.02 ± 1.41 days	5.06 ± 1.80 days		p < 0.001	
	HLOS	13.12 ± 4.21 days	18.57 ± 5.39 days			
	Ventilation Requirements	Not reported	Not reported		Not reported	
	Mortality Rate	Not specified				
Zhang et al. (22)	ICU LOS	5.5 ± 6.4 days	14.2 ± 6.5 days		p < 0.05	
	HLOS	Shorter HLOS reported in surgical group compared to conservative group			Not reported	
	Ventilation Requirements	11(47.83 %)	24(82.76 %)		p <0.01	
	Mortality Rate	No death reported in either group				
Ağababaoğlu & Ersöz (23)	ICU LOS/ HLOS	Shorter in surgical group compared to conservative group			p < 0.001	
	Ventilation Requirements	Fewer in surgical group compared to conservative group			p < 0.001	
	Mortality Rate	2.94%	20.69%		p = 0.027	
	Griffard et al. (24)	ICU LOS	6.0 days (IQR 4 to 9)	3.5 days (IQR 2 to 9)		p = 0.0217	
HLOS		11.0 days	9.0 days		p = 0.1358	
Ventilation Requirements		No significant difference between the operative and conservative groups in ventilator days			p = 0.641	
Mortality Rate		Not reported				
Jayle et al. (25)	ICU LOS	9.0 ± 4.3 days	12.3 ± 8.5 days		p = 0.076	
	HLOS	21.7 ± 7.8 days	32.3 ± 19.3 days		p = 0.024	
	Ventilation Requirements	74 ± 125 hours	142 ± 224 hours		p = 0.026	
	Mortality Rate	Not reported				
Beks et al. (26)^ c^	ICU	6 (0 – 13) days (FC)	2 (0 – 8) days (FC)		p = 0.638 (FC)	
		0 (0 – 11) days (MRF)	1 (0 – 2) days (MRF)		p = 0.530 (MRF)	
	HLOS	21 (11 – 31) days (FC)	11 (8 – 18) days (FC)		p = 0.820 (FC)	
		12 (9 – 23) days (MRF)	10 (6 – 16) days (MRF)		p = 0.074 (MRF)	
	Ventilation Requirements	3 (0 – 9) days (FC)	0 (0 –7) (FC)		p = 0.624 (FC)	
		Mortality Rate	Not reported		Mortality Rate	
		0 (0 – 9) days (MRF)	0 (0 – 1) days (MRF)		p = 0.365 (MRF)	
	Mortality Rate	Not reported				
Fokin et al. (35)	ICU LOS/ HLOS	Longer in surgically treated patients without FC compared to conservatively managed patients without FC (p < 0.001) Presence of pulmonary contusion did not affect outcome. SSRF patients with FC had comparable outcome to conservatively managed patients with FC			p > 0.3	
	Ventilation Requirements	SSRF patients with FC had comparable outcome to conservatively managed patients with FC			p > 0.3	
	Mortality Rate	Mortality was lower in surgically treated patients compared to conservatively managed patients			Not specified	
Xiao et al. (36)^ c^	ICU	5.5 ± 1.9 days (FC)	6.7 ± 2.1 days (FC)		p = 0.011 (FC)	
		4.3 ± 1.5 days (MRF)	4.2 ± 1.8 days (MRF)		p = 0.425 (MRF)	
	HLOS	16.7 ± 6.1 days (FC)	16.8 ± 5.9 days (FC)		p = 0.937 (FC)	
		10.7 ± 3.4 days (MRF)	10.2 ± 3.8 days (MRF)		p = 0.067 (MRF)	
	Ventilation Requirements	20.0% (FC)	20.0% (FC)		p = 1.000 (FC)	
		2.0% (MRF)	12.9% (MRF)		p = 0.732 (MRF)	
	Mortality Rate	4.4%	4.4%		p = 1.000	
		0.9%	1.1%		p = 0.704	
Majeed et al. (37)	ICU LOS	Not reported	Not reported		Not reported	
	HLOS	Mean 23.17 days	Mean 20.89 days		p = 0.55	
	Ventilation Requirements	Mean 19.71 days	Mean 24.18 days		p = 0.12	
	Mortality Rate	No deaths reported	Two deaths reported		Not specified	
Acker et al. (38)^ d^	ICU LOS	21.25 ± 1.4 days	15.43 ± 1.6 days		p = 0.13	
	HLOS	30.67 ± 1.4 days	35.3 ± 4.9 days		p = 0.64	
	Ventilation Requirements	22.2 ± 2.3 days	25.2 ± 4.2 days		p = 0.18	
	Mortality Rate	No statistical difference reported				
Prins et al. (39)^ d^	ICU LOS	6 days	10 days		p < 0.001	
	HLOS	10 days	15 days		p < 0.001	
	Ventilation Requirements	4 days	8 days		p < 0.001	
	Mortality Rate	No difference in mortality rate between groups				
Otaka et al. (40)^ d^	ICU LOS	Not reported	Not reported		Not reported	
	HLOS/Ventilation Requirements	Earlier surgical rib fixation was associated with shorter outcome compared with non-operative management within 3 days after admission No such association for later surgical rib fixation			Not reported	
	Mortality Rate	No significant differences between the groups in all-cause 28-day in-hospital mortality (p = 0.40)				
Su et al. (41)^ d^	ICU LOS	Median = 123 hrs	Median = 230 hrs		p = 0.004	
	HLOS	Median = 12 days	Median = 18 days		p = 0.03	
	Ventilation Requirements	Median = 36 hrs	Median = 90 hrs		p = 0.03	
	Mortality Rate	0%	11.8%		p = 0.10	

**Table 3:** Secondary outcomes – (a) Outcome between operative and conservative management in patients with FC versus MRF. (b) Outcomes pertaining to surgical timing (i.e., <3 days vs. > 3 days) as opposed to surgical versus conservative management. *Outcomes among non-FC patients only. **Outcomes among FC patients only. SSRF - surgical stabilization of rib fractures, FC - flail chest, MRF - multiple rib fractures, NPS - numeric pain score

Study	Secondary Outcome	SSRF	Conservative		P-value	
Tarng et al. (6)	Postoperative Pain Level	Not reported				
	Postoperative Complications	Not reported				
Wijffels et al. (8)	Postoperative Pain Level	Not reported				
	Postoperative Complications	Pneumonia (35%)		Pneumonia (57%)	p = 0.126	
		Respiratory insufficiency (4.0%)		Respiratory insufficiency (13%)	p = 0.517	
		Empyema (0%)		Empyema (2%)	p = 1.000	
		Delirium (13%)		Delirium (36%)	p = 0.076	
Granetzny et al. (10)	Postoperative Pain Level	Not reported				
	Postoperative Complications	Chest infection (10%)		Chest infection (50%)	p = 0.014	
		Empyema (5%)		Empyema (10%)	Non-significant	
		Pulmonary embolism (0%)		Pulmonary embolism (5%)	Non-significant	
		Mediastinitis (10%)		Mediastinitis (0%)	Non-significant	
		Wound infection (10%)		Wound infection (0%)	Non-significant	
		Chest wall deformity (5%)		Chest wall deformity (45%)	p = 0.008	
		Scoliosis (0%)		Scoliosis 25%)	p = 0.047	
		No complications (65%)		No complications (40%)	Non-significant	
Qiu et al. (11)	Postoperative Pain Level	Not reported				
	Postoperative Complications	Pulmonary infection (4.62%)		Pulmonary infection (16.95%)	p = 0.025 *	
		Chest wall deformity (14.29%)		Chest wall deformity (64.71%)	p = 0.002	
Jian et al. (13) **	Postoperative Pain Level	No statistical difference reported				
	Postoperative Complications	No statistical difference reported				
Tanaka et al. (16)	Postoperative Pain Level	12 months follow-up (39%)		12 months follow-up (89%)	p < 0.05	
	Postoperative Complications	Pneumonia at 21 days post-injury (22%)		Pneumonia at 21 days post-injury (90%)	p < 0.05	
Marasco et al. (17)	Postoperative Pain Level	Not specifically assessed				
	Postoperative Complications	Pneumonia (48%)		Pneumonia (74%)	p = 0.07	
Pieracci et al. (18)	Postoperative Pain Level	NPS on hospital day 7 (4.7)		NPS on hospital day 7 (6.3)	p < 0.01	
		NPS at week 2 follow up (2.9)		NPS at week 2 follow up (4.5)	p < 0.01	
		NPS at week 4 follow up (2.4)		NPS at week 4 follow up (3.3)	p < 0.03	
		NPS at week 8 follow up (1.5)		NPS at week 8 follow up (3.3)	p < 0.02	
	Postoperative Complications	Pneumonia (2.0%)	Pneumonia (6.7%)		p = 0.37	
		Pleural space complications (0%)	Pleural space complications (10.2%)		p = 0.02	
Taghavi et al. (19)	Postoperative Pain Level	Not reported				
	Postoperative Complications	ARDS (3.1%)	ARDS (1.0%)		p < 0.001	
Farquhar et al. (20)	Postoperative Pain Level	Not reported				
	Postoperative Complications	Pneumonia (63%)	Pneumonia (22%)		p = 0.004	
		No significant differences in long-term complications, such as chest pain or dyspnea between groups				
Liu et al. (21)						Postoperative Pain Level	Lower pain levels in surgical group vs. conservative group at 24 hrs, 48 hrs, and 72 hrs postop			Not reported	
	Postoperative Complications	Displacement after treatment (n = 1)		Displacement after treatment (n = 2)	p = 0.475	
		Atelectasis (n = 10)		Atelectasis (n = 17)	p = 0.046	
		Delayed hemothorax (n = 7)		Delayed hemothorax (n = 16)	p = 0.012	
Zhang et al. (22)	Postoperative Pain Level	Not reported				
	Postoperative Complications	Respiratory complications (30.43%)		Respiratory complications (75.86)	p <0.005	
		Thoracic deformity (0.0%)		Thoracic deformity (41.38%)	p <0.005	
Ağababaoğlu & Ersöz (23)	Postoperative Pain Level	Lower vs. conservatively managed patients	Higher vs. surgically managed patients		p = 0.0038 vs. p = 0.044	
	Postoperative Complications	No difference in the prevalence of pneumonia (p = 0.315), pulmonary contusion (p = 0.534), or sepsis (p = 0.189) between the two groups				
Griffard et al. (24)	Postoperative Pain Level	Not reported				
	Postoperative Complications	No difference in the prevalence of pneumonia (p = 0.1416) or severe ARDS (p = 0.999) between the two groups				
Beks et al. (26) ^a^	Postoperative Pain Level	Not reported				
	Postoperative Complications	Pneumonia (4.8%) (FC)		Pneumonia (5.6%) (FC)	p = 0.871 (FC)	
		Pneumonia (7.4%) (MRF)		Pneumonia (5.0%) (MRF)	p = 0.114 (MRF)	
Khandelwal et al. (28)	Postoperative Pain Level	Patients undergoing SSRF with plating had reduced pain vs. patients receiving conservative treatment alone at 5, 15, and 30 days postop			p < 0.000	
	Postoperative Complications	Not reported				
Zhang et al. (29)	Postoperative Pain Level	Patients undergoing SSRF with plating had reduced pain vs. patients receiving conservative treatment with analgesia			p < 0.001	
	Postoperative Complications	Not reported				
Fokin et al. (35)	Postoperative Pain Level	Not reported				
	Postoperative Complications	Not reported				
Xiao et al. (36)^ a^	Postoperative Pain Level	Not assessed				
	Postoperative Complications	Pneumonia (28.9%) (FC)	Pneumonia (31.1%) (FC)		p = 0.818	
		Pneumonia (24.3%) (MRF)	Pneumonia (24.9%) (MRF)		p = 0.861	
Majeed et al. (37)	Postoperative Pain Level	Intervention group reported less severe postoperative pain when compared with control group			p = 0.032	
	Postoperative Complications	Restricted shoulder movement, hyperesthesia, persistent pain	ARDS and chronic pain pneumonia		Not reported	
Acker et al. (38)^ b^	Postoperative Pain Level	Not reported				
	Postoperative Complications	Not reported				
Prins et al. (39)^ b^	Postoperative Pain Level	Not reported				
	Postoperative Complications	Not reported				
Otaka et al. (40)^ b^	Postoperative Pain Level	Not reported				
	Postoperative Complications	Not reported				

Limitations

With the rise of rib fixation, a surge in rib fixation systems has also occurred. Examples include intramedullary nails or splints, Kirschner wires, plating, and Judet struts. However, our paper focused primarily on the efficacy of the plating system compared to the current standard of care. Therefore, the outcomes reported herein are not necessarily generalizable to other forms of surgical stabilization excluded from this paper. Which system is best, remains to be seen. Additionally, our findings addressed the overall use of rib plating and did not aim to distinguish between the preferences or success rates of various manufacturer products (i.e., MatrixRIB vs. RibLoc vs. Stratos). Future research comparing the efficacy of these stabilization systems is necessary to better demonstrate superiority in clinical outcomes.

Another limitation is that our search of the literature was limited to PubMed, Google Scholar, and PLOS One search engines which may have limited the results reported in our paper compared to if additional search engines were utilized. Furthermore, only full-text articles with free access were considered for inclusion which narrowed the number of potentially relevant articles that would otherwise have met our inclusion criteria. Likewise, except for an article by Wijffels et al., we excluded over 20 case reports/case series as findings reported in these sources could have low external validity and thus not be helpful in establishing a cause-effect relationship. This could lead to over-interpretation of findings. Additionally, most of the studies referenced in this paper consisted of smaller sample sizes which decrease the power of statistical comparisons and may account for type II errors. Other considerations affecting the outcomes of rib plating are whether chest wall injuries occurred in isolation or polytrauma. We found that certain studies reported a difference in rib plating outcome when traumatic rib injuries occurred in isolation compared to in the settings of comorbidities such as pulmonary contusion, FC, and severe TBIs. Lastly, only eight of the studies included in this review were prospective in nature, therefore, increasing potential sources for bias and confounding herein.

## Conclusions

Our review aimed to underscore the benefits that rib plating offers as an effective approach to managing traumatic rib injuries compared to conservative treatment. Although few reports argue against the efficacy of rib plating, most studies provide substantial evidence favoring surgical treatment. We, therefore, recommend the use of the plating system (where clinically indicated) to stabilize chest wall injuries. It is important to note, however, the context (i.e., type of study, sample size involved, comorbid injuries, etc.) in which these results have been reported. This also draws attention to the need for ongoing research via large, multi-centered RCTs and high-quality observational studies to address gaps in the literature and areas of uncertainty within clinical practice.
